# Unveiling BRAF Mutations in Non-small Cell Lung Cancer: State of the Art and Future Perspectives

**DOI:** 10.1007/s00408-026-00932-9

**Published:** 2026-09-16

**Authors:** Maria Colombino, Panagiotis Paliogiannis, Grazia Palomba, Marina Pisano, Milena Casula, Alessandro Giuseppe Fois, Angelo Zinellu, Giuseppe Palmieri

**Affiliations:** 1 Institute of Genetic & Biomedical Research (IRGB), Unit of Cancer Genetics, National Research Council (CNR), Sassari, Italy; 2 Department of Medicine, Surgery and Pharmacy, University of Sassari, Sassari, Italy; 3 Department of Biomedical Sciences, University of Sassari, Sassari, Italy; 4 Immuno-Oncology and Targeted Cancer Biotherapies, University of Sassari, Sassari, Italy

**Keywords:** BRAF, Lung, Cancer, NSCLC, V600E, Targeted therapy

## Abstract

**Background and Purpose:**

Lung cancer is the most frequently diagnosed malignancy worldwide in recent decades, representing the leading cause of cancer-related mortality globally. Lung cancer is mainly divided into two types, non-small cell lung cancer (NSCLC) and small cell lung cancer (SCLC), with NSCLC being the most common, representing about 85% of cases. NSCLC is increasingly recognized to harbor various druggable genetic alterations; among them, activating BRAF mutations are found in approximately 3–4% of NSCLC patients. The objective of this review is to provide a comprehensive overview of the key biological and pathophysiological characteristics of BRAF molecular alterations, their impact on clinical practice, and emerging insights from translational research.

**Methods:**

We performed a search of the PubMed database on April 22, 2026. A review of retrieved literature related to BRAF mutations and their clinical implications in NSCLC was completed. The Catalogue of Somatic Mutation in Cancer (COSMIC) database and the NCCN-NSCLC Guidelines were evaluated.

**Results:**

Functional classification of BRAF genetic alterations, molecular assays for detection of BRAF gene mutation, advancements of targeted therapies and immunotherapy for BRAF‑mutant NSCLC, and treatment resistance mechanisms were described. Several ongoing studies currently evaluating novel agents with broader biological activity and various drug combinations were reported. Emerging strategies—including next-generation MAPK inhibitors, ADCs, and engineered cellular therapies—were discussed, offering promising avenues to enhance efficacy, overcome resistance, and expand therapeutic options for these patients.

**Conclusion:**

Despite limited clinical data, particularly in first line and neoadjuvant settings, remain major challenges, addressing critical questions will be useful to optimize the management of BRAF-mutant NSCLC.

## Introduction

 Lung cancer has remained the most frequently diagnosed malignancy worldwide in recent decades, accounting for approximately 2.5 million new cases (12.4% of all cancers) and continuing to represent the leading cause of cancer-related mortality globally, with an estimated 1.8 million deaths (18.7%) reported in 2022 [1, 2]. Although more than 50 histomorphological subtypes of lung cancer have been described, the disease is broadly classified into two principal categories: non-small cell lung cancer (NSCLC) and small cell lung cancer (SCLC). Among these, NSCLC is by far the most prevalent, accounting for approximately 85% of all lung cancer cases [3]. NSCLC is further subdivided into three major histological subtypes: adenocarcinoma (ADC), which represents roughly 32–40% of all lung cancers; squamous cell carcinoma (SCC), accounting for approximately 20–30%; and large cell carcinoma (LCC), which comprises about 3–15% of cases [4, 5].

Although histopathological classification remains widely used for the morphological characterization of lung cancer, accumulating evidence indicates that it is increasingly insufficient to guide therapeutic decision-making. Comprehensive molecular profiling studies have demonstrated that lung cancer initiation and progression are primarily driven by genetic alterations that activate oncogenes or inactivate tumor suppressor genes, thereby disrupting key signaling pathways that regulate cell proliferation, survival, and metastasis [6, 7]. These molecular events ultimately promote tumor initiation, progression, and dissemination [8]. Importantly, ADC and SCC, while both classified within the broader category of NSCLC, are now recognized as highly heterogeneous diseases at the molecular level. Each histological subtype encompasses multiple molecularly defined subgroups characterized by distinct somatic driver mutations and genomic alterations [7, 9]. This growing understanding has enabled a more refined stratification of patients into biologically and clinically relevant subgroups based on specific oncogenic alterations [10]. These advances have profoundly reshaped the clinical management of lung cancer by enabling the development of more precise diagnostic frameworks and targeted therapeutic strategies, ultimately paving the way for personalized treatment approaches tailored to the molecular profile of each individual tumor.

Currently, a growing number of genetically actionable driver alterations have been identified in NSCLC, including: (a) EGFR, KRAS, BRAF, and ERBB2 (HER2) gene mutations; (b) ALK, ROS1, RET, NRG1, and NTRK gene fusions encoding aberrant transcripts; and (c) MET exon 14 skipping mutations preventing MET degradation and subsequent receptor accumulation-driven oncogenic activity [11]. Each of such alterations contributes to distinct oncogenic signaling pathways and represents a clinically druggable target [12–14]. Routine molecular testing for these alterations is strongly recommended by both national and international guidelines to guide targeted therapy selection and optimize patient outcomes [15, 16]. In addition, assessment of programmed death-ligand 1 (PD-L1) expression is advised to identify patients eligible for immune checkpoint inhibitors (ICIs), which have been shown to improve survival in patients without oncogene-addicted tumors [17]. PD‑L1 testing is currently the most widely validated biomarker for guiding the use of PD‑1/PD‑L1 axis blockade, with higher expression associated with improved responses to single‑agent ICIs in selected populations, although responses can also occur across PD‑L1 levels [17, 18]. These advances in molecular diagnostics and immunotherapy have markedly expanded the therapeutic armamentarium available to modern oncologists, leading to significant improvements in clinical outcomes for patients with NSCLC.

Activating mutations in BRAF are identified in approximately 3–4% of patients with NSCLC [19–21]. Although relatively uncommon, these alterations define a biologically and clinically distinct subset of NSCLC patients who may derive benefit from both targeted therapies and immune-based approaches. Several anti-BRAF therapeutic regimens are currently approved for clinical use, and numerous additional strategies are being evaluated in ongoing clinical trials. The objective of this review is to provide a comprehensive overview of the key biological and pathophysiological characteristics of BRAF molecular alterations, their impact on contemporary clinical practice, and emerging insights from translational research. Furthermore, we highlight current challenges and critical questions that must be addressed in future studies to optimize the management of BRAF-mutant NSCLC.

## BRAF Biology and Molecular Alterations

BRAF encodes a serine/threonine kinase that is a member of the RAF family of kinases, comprising ARAF, BRAF, and CRAF, and functions as a key regulator of the mitogen-activated protein kinase (MAPK) signaling pathway, where it acts by phosphorylating and activating the mitogen‐activated extracellular signal regulated kinase (MEK), which in turn is responsible for extracellular signal‐related kinase (ERK) phosphorylation and activation, playing a central role in controlling cell growth, differentiation, and survival [22]. Although the three family members share a similar structural architecture, they differ in their ability to activate the downstream substrate MEK, and their catalytic activation critically depends on dimer formation through either homo- or heterodimerization [23, 24]. Oncogenic BRAF mutations result in constitutive activation of this signaling cascade, ultimately driving aberrant and sustained cell proliferation [22].

Under physiological conditions, the MAPK signaling pathway is initiated by the binding of extracellular growth factors to receptor tyrosine kinases (RTKs), such as EGFR, a transmembrane receptor localized at the cell surface. Ligand binding promotes EGFR dimerization and autophosphorylation, which in turn triggers the activation of KRAS, a small monomeric GTPase that becomes active upon binding GTP. Activated KRAS subsequently recruits and promotes the phosphorylation and activation of BRAF, thereby initiating a kinase cascade that culminates in the activation of ERK2; once activated, ERK2 translocates to the nucleus, where it phosphorylates transcription factors including c-JUN and ELK1, ultimately regulating the expression of genes involved in cell-cycle progression and proliferation [22, 25–27]. Typically, the MAPK signaling pathway is attenuated through multiple negative feedback mechanisms once the signal has been propagated, ensuring tight regulation of pathway activity. However, as mentioned before, activating mutations in BRAF result in constitutive activation of the MAPK cascade, leading to persistent downstream signaling independent of upstream regulatory inputs [22, 28]. This sustained pathway activation drives uncontrolled cell growth and proliferation, thereby contributing to oncogenesis in several malignancies, including NSCLC and melanoma.

To date, more than 200 distinct BRAF mutations have been identified across a wide range of cancer types, predominantly affecting two critical regions of the kinase domain: the activation segment (exon 15) and the glycine-rich P-loop (exon 11) [28]. Although several of these alterations markedly increase BRAF kinase activity, the functional consequences of many variants remain incompletely characterized, posing challenges for the development of effective targeted therapeutic strategies. The most common activating alterations are point mutations affecting valine at position 600 within exon 15, which result in constitutive activation of the BRAF kinase [29]. Among these, the V600E substitution, where valine is replaced by glutamic acid, represents the most common BRAF mutation, with the second most frequent variant constituted by the V600K mutation, in which valine is replaced by lysine [30–31]. The V600E variant accounts for up to 50% of all BRAF mutations in NSCLC, with the remaining BRAF mutated cases presenting substitutions at this residue (V600K, occurring in less than 10% of BRAF mutants, and V600R/D/M, altogether observed in up to 5% of cases) or mutations in other than V600 residue (e.g., G464, G469, K601, and L597), each of them presenting a considerably lower frequency [30, 32]. Structurally, the V600E substitution disrupts the hydrophobic interaction between the P-loop and the activation segment of the kinase domain, thereby destabilizing the inactive conformation of BRAF, increasing kinase activity and resulting in sustained ERK phosphorylation within the MAPK pathway, ultimately promoting uncontrolled cellular proliferation [32, 33].

Overall, non‑V600 mutations of the BRAF gene account for a significant minority of BRAF‑altered tumors, representing roughly 35% of all BRAF mutations across diverse cancer types in large genomic cohorts [33]. In contrast, oncogenic BRAF gene fusions are rare in unselected solid tumors, occurring in less than 1% of cases, but are notably enriched in specific histologic subtypes such as pilocytic astrocytomas, Spitzoid melanomas, and pancreatic acinar cell carcinomas [34]. Given the heterogeneity of BRAF mutations and their distinct functional consequences, accurate classification is essential for understanding their roles in oncogenesis and their impact on therapeutic response. Here, we provide a detailed examination of the current classification of BRAF mutations.

## Functional Classification of BRAF Genetic Alterations

**Fig. 1:**
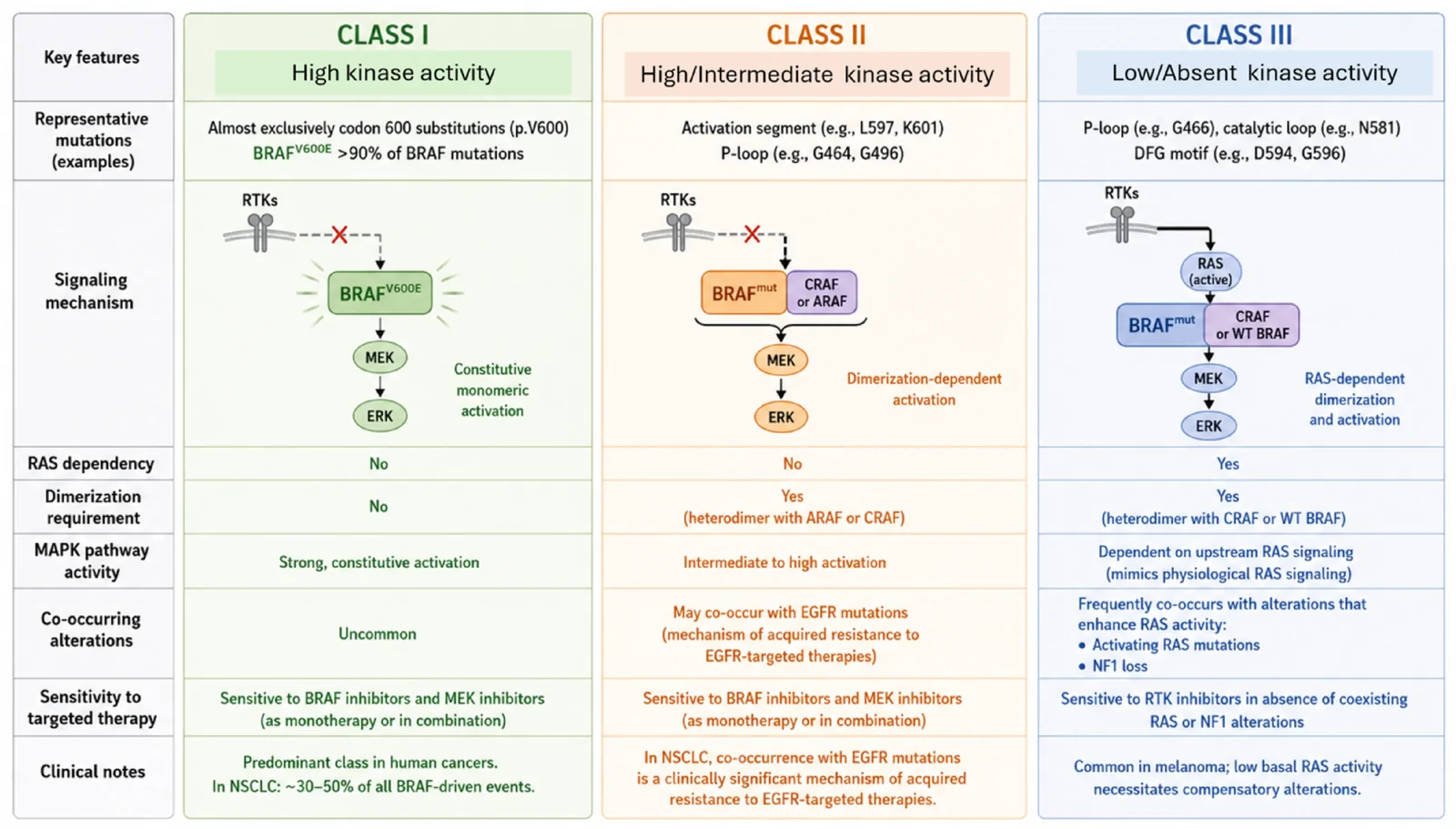
Biochemical features, signalling mechanisms, and therapeutic implications of the BRAF mutation classes. RTKs, receptor tyrosine kinases; WT, wild-type

A major challenge in developing effective therapies for BRAF‑mutant cancers stems from the extensive heterogeneity of mutations within the BRAF gene. This diverse mutational landscape results in distinct biochemical behaviors: some alterations drive robust BRAF activation, whereas others exhibit intermediate or context-dependent effects [35]. BRAF mutations are categorized into three classes based on key biochemical and signaling characteristics, including kinase activity, dimerization status, and RAS dependency (Fig. 1) [35, 36]. A detailed understanding of these functional classes is essential for predicting responses to MAPK-targeted therapies and for guiding the design of more precise and effective treatment strategies.

*Class I BRAF mutations* are the predominant alterations across human cancers, almost exclusively involving codon 600 substitutions (p.V600). The BRAFV600E variant is the most frequent, representing > 90% of BRAF mutations [32, 35, 36]. In NSCLC, Class I mutations comprise approximately 30–50% of all BRAF-driven events and are characterized by strong constitutive activation of BRAF kinase, driving persistent MAPK pathway signaling. This sustained signaling occurs independently of upstream RAS activation and RAF dimerization (ARAF or CRAF), and confers sensitivity to BRAF and MEK inhibitors, administered either as monotherapy or in combination [37–39].

*Class II BRAF mutations* frequently localize to the activation segment of the kinase domain (e.g., L597, K601) or the P-loop (e.g., G464, G496) and exhibit intermediate to high kinase activity [35–39]. These mutations signal independently of upstream RAS activation but require dimerization with other RAF proteins to sustain MAPK pathway activation. Class II mutations remain sensitive to BRAF and MEK inhibitors, administered as monotherapy or in combination, and, when co-occurring with EGFR mutations, represent a clinically significant mechanism of acquired resistance to EGFR-targeted therapies [36].

*Class III BRAF mutations* are typically localized to the P-loop (e.g., G466), catalytic loop (e.g., N581), and DFG motif (e.g., D594, G596) and exhibit low or absent intrinsic kinase activity. These mutations rely on upstream RAS activation to drive MAPK signaling through RAS-dependent heterodimerization with CRAF or wild-type BRAF, effectively mimicking physiological RAS signaling [37–39]. Class III mutations frequently co-occur with alterations that enhance RAS activity, such as activating RAS mutations or NF1 loss [36]. This co-occurrence is particularly common in melanoma, where low basal RAS activity necessitates compensatory mechanisms. The dependence of Class III mutations on upstream signaling renders them highly sensitive to RTK inhibitors in the absence of coexisting RAS or NF1 alterations [36, 40].

BRAF deletions and fusions represent less common but biologically significant alterations in cancer, driving tumorigenesis through distinct effects on the MAPK pathway and representing potential therapeutic targets. In-frame deletions within the α-C helix of the kinase domain (e.g., L485_N486) have been reported, paralleling EGFR exon 19 deletions. Mechanistically, these deletions enhance kinase activity independently of RAS signaling and, similarly to Class II mutations, promote constitutive MAPK pathway activation via homodimer formation [41–43]. Additional N-terminal deletions (e.g., NTD-BRAF, involving exons 2 to 9) have also been identified, activating MAPK signaling by removing autoinhibitory regions, which may contribute to resistance to therapies such as vemurafenib [42]. BRAF fusions involve loss of the N-terminal autoinhibitory domain while retaining the kinase domain, leading to constitutive MAPK activation through formation of active heterodimers, such as BRAF-CRAF dimers [43, 44]. The most common fusion partner is AGK. Mechanistically, BRAF deletions and fusions resemble Class II mutations and are observed in a small subset of NSCLC.

Rare additional alterations, such as amino acid insertions at codon 599, can perturb the P-loop structure and enhance kinase activity, while chromosomal translocations disrupting the inhibitory domain similarly result in MAPK overactivation, mimicking BRAF fusions. These mechanisms remain under investigation but are thought to contribute to tumorigenesis and therapeutic resistance across multiple cancer types [38, 39, 45].

## BRAF Genetic Alterations in Cancer

The first evidence linking BRAF gene mutations to human cancer was reported in 2002, establishing BRAF as a key oncogenic driver within the MAPK signaling pathway [20]. Subsequent studies have documented variable frequencies of BRAF alterations across different malignancies, and current estimates indicate that activating BRAF mutations occur in approximately 8% of all solid tumors [46]. The distribution and mutational spectrum of BRAF across human cancer types can be examined using data from the Catalogue of Somatic Mutations in Cancer (COSMIC), version 90, a comprehensive repository of somatic alterations identified in tumor sample [47]. Analysis of 57,831 records derived from targeted sequencing as well as whole-genome and whole-exome studies in which the BRAF coding region was screened identified 638 pathogenic BRAF mutations across 35 distinct human cancer tissues after excluding cases with unknown primary origin or incomplete mutation annotation [48]. Comparable patterns in the distribution of BRAF mutations across tumor types have also been reported in datasets from The Cancer Genome Atlas (TCGA), comprising multiple large-scale genomic studies of human cancers [49].

BRAF mutations are among the most prevalent genetic alterations in thyroid malignancies, particularly in papillary thyroid carcinoma (PTC), where they play a central role in tumor initiation and progression [50]. The most common alteration, the BRAF V600E mutation, which occurs in approximately 40–60% of PTC cases and has been consistently associated with more aggressive clinicopathologic features, including extrathyroidal extension and lymph node metastasis [51]. Moreover, BRAF-mutant tumours often demonstrate reduced responsiveness to radioiodine therapy, highlighting the clinical relevance of targeted therapeutic strategies directed against BRAF or downstream MAPK signaling components [52].

After thyroid tumours, malignant melanoma is the cancer type that exhibits the highest frequency of BRAF gene mutations. Approximately 40–50% of melanomas harbour BRAF mutations, most frequently affecting codon 600 in exon 15 of the gene [53]. Again, the most prevalent variant is BRAF V600E, which accounts for most BRAF-mutant melanomas, except for uveal melanomas in which BRAF mutations are exceedingly rare [54, 55]. The identification of BRAF mutations has significant therapeutic implications, as patients with BRAF-mutant melanoma substantially benefit from targeted therapies with BRAF and MEK inhibitors, which have substantially improved outcomes in advanced disease.

Beyond PTC and melanoma, BRAF mutations are detected with notable frequency in several additional malignancies, most prominently colorectal cancer, where activating mutations occur in approximately 8–12% of cases and are associated with distinct clinical and molecular features [56]. In patients with microsatellite instability (MSI), the frequency of BRAF mutations may reach up to 60% of cases [57]. In addition, BRAF mutations occur in hairy cell leukaemia, where the V600E substitution is present in the vast majority of cases and represents a defining molecular hallmark of the disease [58]. Lower, yet still clinically relevant, frequencies of BRAF mutations have also been reported in gliomas, cholangiocarcinomas, and certain pediatric brain tumors [48].

## BRAF Genetic Alterations in NSCLC

As previously mentioned, activating mutations in BRAF are identified in approximately 3–4% of patients with NSCLC [17–19]. Class I alterations account for approximately 30–50% of all BRAF mutations. As a result, a substantial proportion of BRAF-mutant NSCLC, estimated at 50–80%, harbor non-V600 variants [59, 60]. These alterations are commonly referred to as “unusual” or “rare” BRAF mutations and predominantly fall within the functional Class II and Class III categories. Class II mutations are substitutions other than V600E (e.g., G464, G469, K601, and L597) described in up to 25% of NSCLC. These mutations, when coexisting with EGFR mutations, are considered an important mechanism of acquired resistance to EGFR inhibitors. The reported co-occurrence rate of EGFR and BRAF mutations in NSCLC varies considerably across studies, ranging from 12 to 31% [19, 61], while concurrent activating RAS mutations are observed in approximately 22% of cases with Class III BRAF alterations like G466, S457, N581, and D594 [36, 39, 40].

Among the individual BRAF mutations detected in NSCLC, the oncogenic V600E variant is the most prevalent, accounting for approximately 40% of all BRAF alterations. This is followed by mutations at codons G469 and G466, which account for approximately 22% and 11% of cases, respectively [17–19, 62, 63]. These mutations generally do not overlap with EGFR and METes14 skipping mutations, as well as with RET, ALK and ROS1 rearrangements [64, 65]. Beyond point mutations, BRAF fusions are rare activating events in NSCLC (< 1%) that drive oncogenic signaling through the formation of RAS-independent BRAF dimers with elevated kinase activity, most frequently involving fusion partners such as TRIM24 and SND1 [66].

Regarding NSCLC histological subtypes, it has been widely demonstrated that BRAF mutations occur predominantly in adenocarcinomas, often showing micropapillary morphology. Early studies using real time polymerase chain reaction (PCR) assays targeting BRAF exons 11 and 15 reported BRAF mutations in approximately 5% of lung adenocarcinomas, with the V600E variant accounting for nearly 60% of cases [67]. Subsequent whole-exome sequencing analyses suggested a higher prevalence of BRAF alterations (7–8%) [68], while more recent studies analyzing circulating tumor cell-free DNA have reported mutation rates approaching 9%, although V600E represented only a small proportion (~ 2%) of cases [69]. In contrast, BRAF mutations appear to be uncommon in squamous cell lung carcinoma, although the historically limited molecular testing in this subtype should be taken into account [70–71]. Currently, molecular testing is recommended for all patients with advanced stage and metastatic non-squamous NSCLC and may also be considered for those with squamous cell carcinoma, although the prevalence of actionable alterations is lower in squamous cases [72–73].

BRAF-mutant NSCLC generally presents in patients in their sixth or seventh decade of life and does not demonstrate a clear sex or ethnic predominance. However, smoking history appears to differ across mutational classes. Class I alterations are more frequently observed in females, whereas non-V600 mutations are more commonly identified in males and are relatively more prevalent among never-smokers compared with Class II or Class III mutations [12]. Pleural metastases are commonly observed at the time of diagnosis [67]. In addition, one study reported a higher incidence of axillary lymph node metastases in BRAF-mutated NSCLC compared with tumors lacking BRAF alterations [74]. A more recent analysis suggested that BRAF mutations may be associated with higher programmed death-ligand 1 (PD-L1) expression and low-to-intermediate tumor mutational burden (TMB) [75]; however, these findings have not been consistently confirmed in subsequent studies [76].

## Clinical Implications and Current Treatments

### Targeted Therapy

**Fig. 2:**
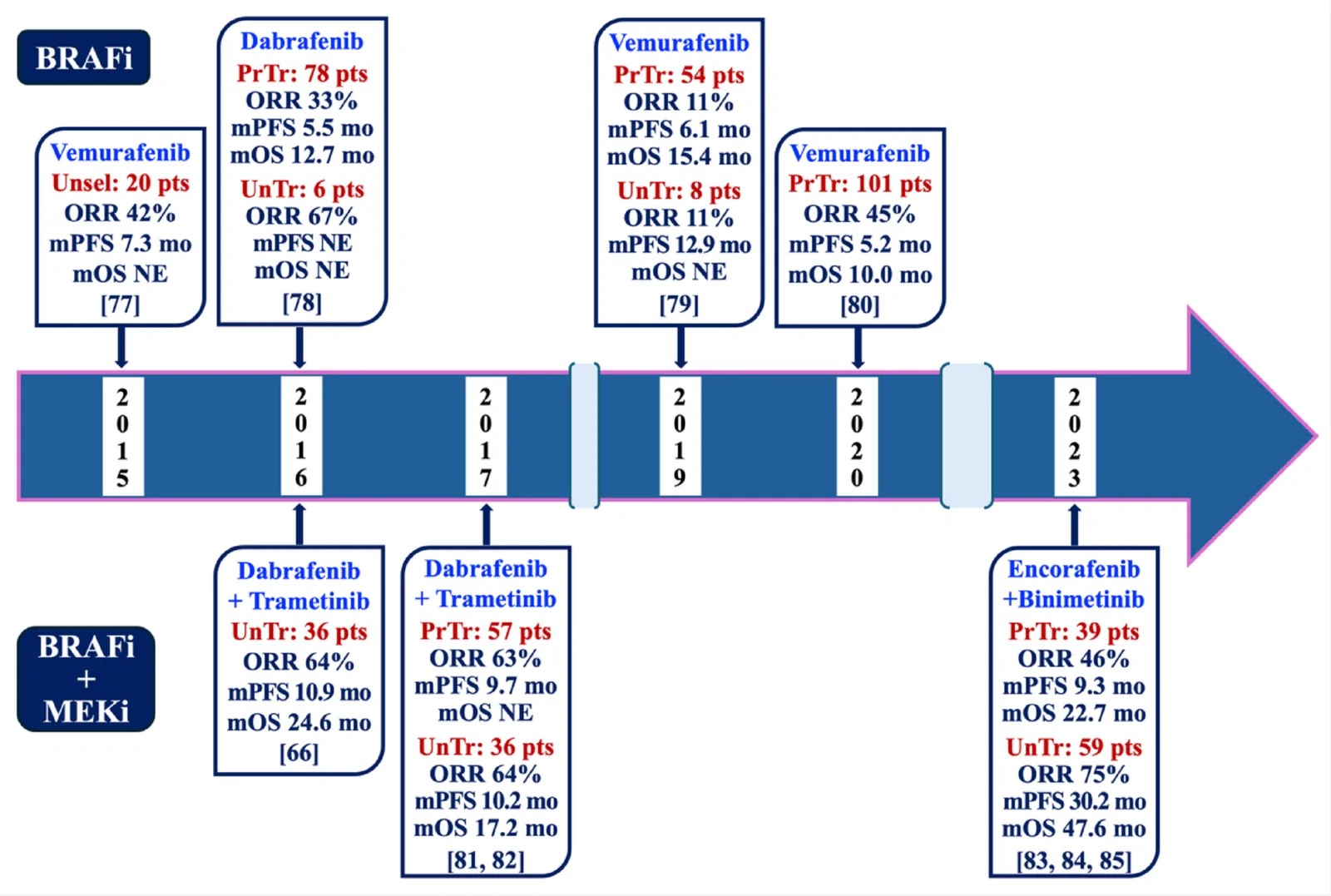
Results from clinical trials based on BRAF inhibitor (BRAFi) alone or combination of BRAF-MEK inhibitors (BRAFi + MEKi) among BRAF-mutated NSCLC patients (pts). ORR, objective response rate; mPFS, median progression-free survival; mOS, median overall survival; NE, not evaluable; PrTr, previously-treated patients; UnTr, untreated/treatment-naive patients; Unsel, patients unselected for previous treatment

The scientific insights discussed thus far have had substantial implications for clinical practice. Two selective inhibitors of the BRAF kinase, dabrafenib and vemurafenib, have been developed and approved for clinical use in patients with BRAF-mutated NSCLC. The former was evaluated in the BRF113928 study (NCT01336634), whereas the latter was investigated in the VE-BASKET and AcSé trials [66, 77–80]. In 2015, a basket study aimed at testing antitumor activity of vemurafenib in nonmelanoma BRAF-V600 mutation-positive cancers showed preliminary efficacy of such a BRAF inhibitor in BRAF-mutated NSCLC patients (nearly all of them were previously treated) [77]. In Fig. 2, the progresses of the anti-BRAF therapy in advanced NSCLC across the years—starting from the efficacy data for vemurafenib in 2015 or dabrafenib in 2016 and moving through the studies leading to the approval of the combinations of dabrafenib-trametinib in 2017 and encorafenib-binimetinib in 2023 [66, 77–85]—are reported.

Vemurafenib and dabrafenib administered as single anti-BRAF agents demonstrated favorable clinical activity; however, their use has also been associated with significant adverse events, partly attributable to the paradoxical activation of the MAPK pathway in BRAF wild-type cells, which led to complications such as the development of secondary cutaneous squamous cell carcinomas and others. Combinations of BRAF and MEK inhibitors (both dabrafenib-trametinib and encorafenib-binimetinib) have been introduced to mitigate some of these toxicities, enhance clinical responses and overcome the development of resistance, and are currently recommended for both first line and subsequent treatment in patients with BRAF V600E–mutated disease, irrespective of performance status [73]. Regarding drug safety, adverse events with BRAF/MEK inhibitors were similar whether used in first or second line of treatment. In Table 1, schematic toxicity profiles for the different BRAFi/MEKi combinations—as in approval trials for NSCLC and, mainly, melanoma [32, 86–88]—are reported.

**Table 1:** Main toxicities associated with the combination of BRAF and MEK inhibitors in BRAF-mutated NSCLC patients

Adverse events	Vemurafenib		Dabrafenib-Trimetinib		Encorafenib-Binimetinib	
	All Grades (%)	Grade ≥ 3 (%)	All Grades (%)	Grade ≥ 3 (%)	All Grades (%)	Grade ≥ 3 (%)
Pyrexia	12	2	50	5	20	4
Fatigue	15	2	20	3	30	2
Nausea	7	1	45	0	45	3
Diarrhea	5	1	30	2	40	4
Vomiting	3	1	30	3	40	4
ALT/AST increase	1	0	1	0	7	5
Photosensitivity	58	5	3	0	4	1
Skin toxicity* (rush)	28	8	18	2	12	0
Arthralgia	18	3	15	1	10	0
Ocular toxicity	2	0	5	1	15	2

ALT alanine aminotransferase, AST aspartate aminotransferase

*For Vemurafenib, cutaneous squamous-cell carcinomas (grade 3, 12%) and keratoacanthomas (grade 3, 6%) were reported

Currently, the combinations approved for clinical use are dabrafenib plus trametinib and encorafenib plus binimetinib. In an open-label, multicenter, phase II trial (NCT01336634), the combination of the BRAF V600E inhibitor dabrafenib and the MEK1/2 inhibitor trametinib demonstrated superior clinical activity compared with dabrafenib monotherapy in previously treated patients with BRAF V600E–mutant NSCLC [66]. Among 57 pretreated patients, the regimen achieved an objective response rate (ORR) of 63.2% (95% CI, 49.3–75.6) and a median progression-free survival (PFS) of 9.7 months. The most common adverse event reported in the trial was pyrexia.

The dabrafenib–trametinib combination was also evaluated as first-line therapy in patients with BRAF V600–mutant NSCLC in the same phase II trial (NCT01336634) [81]. In 36 treatment-naïve patients, the regimen demonstrated substantial antitumor activity, with an ORR of 64% (95% CI 46.2–79.2) and a median PFS of 10.2 months. Long-term follow-up reported a median overall survival (OS) of 17.2 months and a 5-year OS rate of 22% [82].

The combination of encorafenib plus binimetinib was evaluated in patients with BRAF V600E–positive metastatic NSCLC in both first line and previously treated settings in the phase II PHAROS study [83]. In the first analysis (September 22, 2022), the primary end point of objective response rate (ORR) by independent radiology review (IRR) was met in both treatment-naive (*n* = 59; 75%) and previously treated (*n* = 39; 46%) patients [83]. In an updated analysis (April 1, 2024), the ORR was unchanged in both groups [84]. The median duration of response and median PFS by IRR were 40.0 and 30.2 months in treatment-naive patients, respectively, and 16.7 and 9.3 months in previously treated patients, respectively. Median OS was 22.7 months in previously treated patients, while results in treatment-naive patients were reported in a further update with data cut-off on March 14, 2025 [85]. In this report, the median duration of treatment with both encorafenib and binimetinib was 16.3 months in treatment-naive and 5.5 months in previously treated patients. After median follow-up for OS of 52.3 months in treatment-naive patients, median OS was 47.6 months, while the 4-year OS probability was 49%. After median follow-up for OS of 48.2 months in previously treated patients, median OS was 22.7 months, and 4-year OS probability was 31%. In treatment-naive and previously treated groups, 58% and 26% received more than one subsequent systemic anticancer treatment, respectively. Safety profile remained consistent with that in previous analyses. The authors underlined that encorafenib plus binimetinib was associated with the longest mOS reported with targeted treatment in patients with treatment-naive BRAF V600E-mutant metastatic NSCLC [85].

A recent study compared the efficacy and safety of dabrafenib plus trametinib versus encorafenib plus binimetinib using individual patient data from the BRF113928 and PHAROS studies, matched on validated adjustment factors and using weighted Cox proportional hazards models for OS and PFS and logistic regression models for objective response rate, grade 3–4 adverse events, serious adverse events (SAEs) and treatment discontinuation [89]. Compared with dabrafenib plus trametinib, encorafenib plus binimetinib was associated with a statistically significant improvement in PFS (HR 0.47; *p* = 0.01) and non-significant improvements in OS and ORR. Encorafenib plus binimetinib was also associated with a statistically significant reduction in SAEs (OR 0.35; *p* = 0.02), while reductions in grade 3–4 adverse events (OR 0.93; *p* = 0.87) and treatment discontinuations (OR 0.71; *p* = 0.53) were not statistically significant. The authors concluded that encorafenib plus binimetinib was associated with statistically significantly longer PFS and fewer SAEs compared with dabrafenib plus trametinib [89]. Additional targeted agents and therapeutic combinations are currently under investigation, as discussed in the following section.

### Immunotherapy

Occurrence of alterations in driver oncogenes underlaying proliferation and survival of tumor cells—such as mutations in EGFR or BRAF—has been reported to trigger immune evasion through induction of an immunosuppressive tumor microenvironment (TME) [90, 91]. Oncogenic BRAF may contributes to TME changes that promote development of resistance to immune checkpoint inhibitor (ICI)-based immunotherapy; in melanoma, targeting mutated BRAF induces PD-L1 overexpression was found to increase the tumor immunogenicity by promoting T cell infiltration and decreasing immunosuppressive cytokines [92, 93].

Studies showed that NSCLC harboring the BRAF mutation derived more benefit from ICIs compared to other oncogene-addicted (i.e. EGFR/ALK/RET-driven) or unselected cancers, which could be due to elevated TMB and PD-L1 expression in these tumors [94, 95]. Therefore, treatment decisions in patients with BRAF-mutated advanced NSCLC should consider specific clinico-pathological factors, including the extent of smoking history, the levels of PD-L1 expression, and the requirement for corticosteroids to manage brain metastases, which may negatively impact the efficacy of immune checkpoint inhibitors [94, 96, 97]. However, majority of patients with *BRAF*-mutant NSCLC has been reported to present progressive disease as best response to ICI; altogether, median PFS of ICI-based treatments among these patients varies between the different studies but is generally poor (4 months or less) [98]. Although the response rates with ICIs are lower compared to targeted therapies, immunotherapy remains a viable option in subgroups of NSCLC patients with BRAF‐mutant tumor and high PD‐L1 expression or with an active/former smoking history [32].

For NSCLC patients with BRAF mutations exerting intermediate or low kinase activity (class II/III variants), a platinum-based chemotherapy may represent a treatment option in the absence of approved targeted therapies [98]. On this regard, data about the real impact of the different subtypes of BRAF mutations on response to chemotherapy are still lacking. To date, chemotherapy is usually associated with ICI in protocols of chemoimmunotherapy, mainly when PD-L1 expression is present but at intermediate/low levels; however, such a therapeutic strategy appears less effective in NSCLC tumors carrying mutations in driver oncogenes, including BRAF, probably due to the above-described tendency to induce an immune-cold tumor microenvironment [99].

**Fig. 3:**
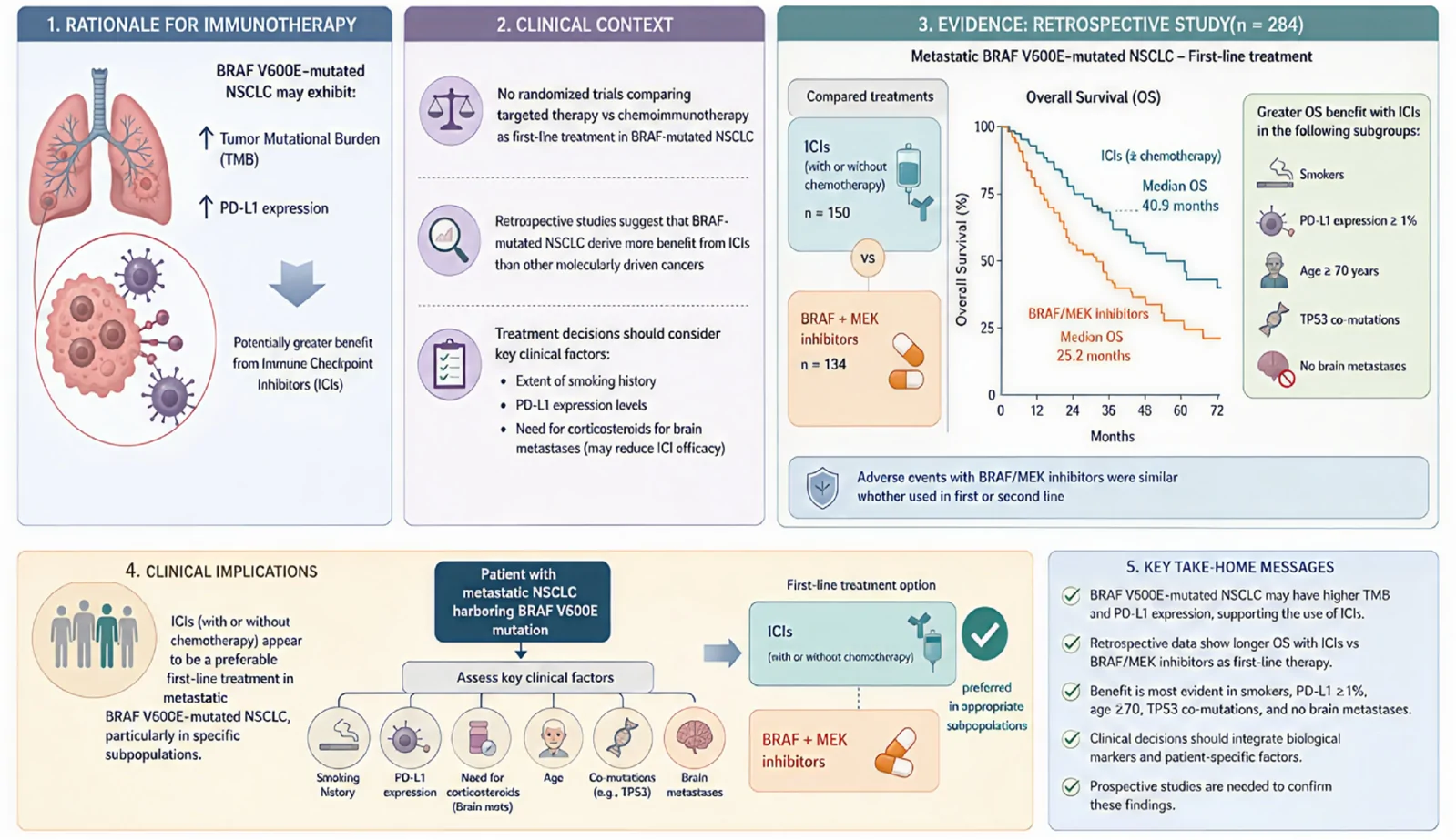
Rationale, evidence, and clinical implications of immunotherapy in advanced NSCLC harboring BRAF V600E mutation. ICIs, immune checkpoint inhibitors; PD-L1, Programmed Death-Ligand 1; TMB, tumor mutational burden; mets, metastases; OS, overall survival

To date, no randomized clinical trials have directly compared targeted therapies with (chemo)immunotherapy as first line treatment for BRAF-mutated NSCLC. A recent retrospective study evaluated 284 patients with metastatic BRAFV600E-mutated NSCLC, comparing first-line PD-1 or PD-L1 inhibitors, with or without chemotherapy, versus BRAF and MEK inhibitors [97]. After a median follow-up of 45 months, ICIs were associated with longer overall survival (40.9 vs. 25.2 months). The survival benefit was more pronounced in specific subgroups, including smokers, patients with PD-L1 expression ≥ 1%, those aged ≥ 70 years, with TP53 co-mutations, and without brain metastases. Overall, ICIs (with or without chemotherapy) appear to be a preferable first-line treatment option in patients with metastatic BRAFV600E-mutated NSCLC, particularly among the above-mentioned specific subpopulations. Although confirmation from prospective studies is awaited, these findings open the way to future research aimed at identifying patient subgroups who may derive benefit from immunotherapy, including a better clarification about the relationships between outcome and specific class of BRAF mutations. In any case, current guidelines recommend considering first line treatment with an ICI–based regimen in selected patients, particularly those with a low tumor burden and/or high PD-L1 expression [73]. All issues regarding the immunotherapeutic approach in BRAF-mutant NSCLC are depicted in Fig. 3.

Despite the lack of randomized clinical trials comparing (chemo)immunotherapy versus targeted therapy with anti-BRAF/MEK as well as the availability of retrospective data only, the upfront treatment approach with the combination of BRAF and MEK inhibitors should be considered as first option when a rapid response and a fast improvement of the symptoms are needed. Since the use of corticosteroids has been associated with a decreased response rate and a shorter PFS in patients with brain metastases treated with ICIs (mainly, when steroids are administered at baseline than on-treatment) [100], the targeted therapy with anti-BRAF/MEK can represent a good option in presence of brain metastases needing high-dose steroids [101, 102].

## Techniques for the Detection of BRAF Genetic Alterations

The clinical implications discussed above have made molecular testing for BRAF gene mutations necessary and recommended in patients with non-small cell lung cancer (NSCLC). Indeed, numerous national and international guidelines currently recommend molecular testing for the detection of these mutations for clinical purposes to enable appropriate treatment planning for patients [14, 30, 103, 104]. Despite potential limitations related to costs and turnaround times, the National Comprehensive Cancer Network (NCCN) panel currently recommends comprehensive molecular profiling for patients with advanced or metastatic NSCLC to identify the most appropriate therapeutic strategies [14]. This recommendation reflects the need to address the frequent limitations in tissue sample availability as well as the growing number of targeted therapies currently available. For these reasons, multiplex DNA sequencing approaches, such as next-generation sequencing (NGS), are currently favored over traditional methods including Sanger sequencing and quantitative PCR, while immunohistochemistry testing with the monoclonal antibody VE1 for the BRAF V600E mutation can be employed only after strictly adequate validation [32, 73]. Comprehensive genomic profiling performed with NGS can be implemented both at initial diagnosis and at the time of disease progression, enabling the identification of potential mechanisms of resistance to targeted therapies.

Regarding this latter aspect and considering the impracticality of performing multiple tissue biopsies due to their invasiveness, important advances have been achieved with the introduction and implementation of liquid biopsy. The analysis of circulating tumor DNA (ctDNA) obtained from liquid biopsy samples represents a non-invasive alternative for the identification of somatic mutations and is currently recommended by clinical guidelines for monitoring targeted therapies in patients with advanced and metastatic NSCLC [73, 105–108]. Testing of ctDNA can reduce the occurrence of false-positive results associated with fixation-related degradation and deamination of nucleic acids extracted from formalin-fixed, paraffin-embedded tissue samples. Moreover, ctDNA analysis may better capture overall tumor dynamics, as it reflects systemic tumor activity and is less affected by the intratumoral heterogeneity that often limits the representativeness of single-site tissue biopsies. Conversely, the concentration and purity of tumor-derived nucleic acids in the bloodstream are frequently low, which can pose several technical challenges for their accurate detection and analysis [107, 108]. Liquid biopsy can serve as a noninvasive alternative to conventional tissue biopsy when tumor material is unavailable, such as in patients with significant comorbidities, poor performance status, inconclusive tissue results, or lesions that are not readily accessible for sampling, as well as for treatment planning, patient stratification for enrollment in clinical trials and the monitoring of therapeutic response with potential identification of emerging resistance mechanisms [32, 109, 110].

From a methodological standpoint, single-gene digital droplet PCR and multiplex NGS can be applied to detect BRAF mutations in blood samples via ctDNA sequencing. The choice of cfDNA assay depends on multiple factors, including the intended clinical or research application, detection sensitivity, multiplexing capability, cost, and turnaround time. The International Society for Liquid Biopsy (ISLB) suggests detection of mutations for targeted therapy selection and resistance monitoring with targeted NGS panels or comprehensive genomic profiling facilitating simultaneous assessment of multiple biomarkers or with digital PCR which offers a precise, cost-effective option for targeted mutations, though its limited genomic coverage restricts broader applications [108].

## Issues for Research: Drug Resistance

One of the most significant challenges associated with targeted therapies, not only in NSCLC and BRAF-directed treatments but more broadly, is the development of resistance, which can be either primary or acquired. In NSCLC, response rates to BRAF inhibitor monotherapy are much lower than in melanoma, suggesting a higher occurrence of primary resistance; in responsive patients, clinical outcome is then encountering the development of acquired resistance [32]. As largely demonstrated for melanoma in past years, response to BRAF/MEK inhibitors is not durable in vast majority of BRAF-mutated patients and resistance to the target therapy usually develops in about 1 year or less from the initiation of treatment [111]. In NSCLC, the median PFS reported in trials may vary from 5 to 7 months in previously treated patient undergoing treatment with BRAF inhibitor alone to up to 30 months in treatment-naive patients undergoing therapy combining BRAF and MEK inhibitors (about 11 months for Dabrafenib-Trametinib and 30 months for Encorafenib-Binimetinib) (see Fig. 2). Regarding the mechanisms of resistance to BRAF/MEK inhibitors, despite a limited amount of evidence reported for BRAF-mutated NSCLC patients undergoing such a targeted therapy [112], the heterogeneous events leading to the development of the acquired resistance can be considered as quite identical to those previously described in melanoma [111].

**Fig. 4:**
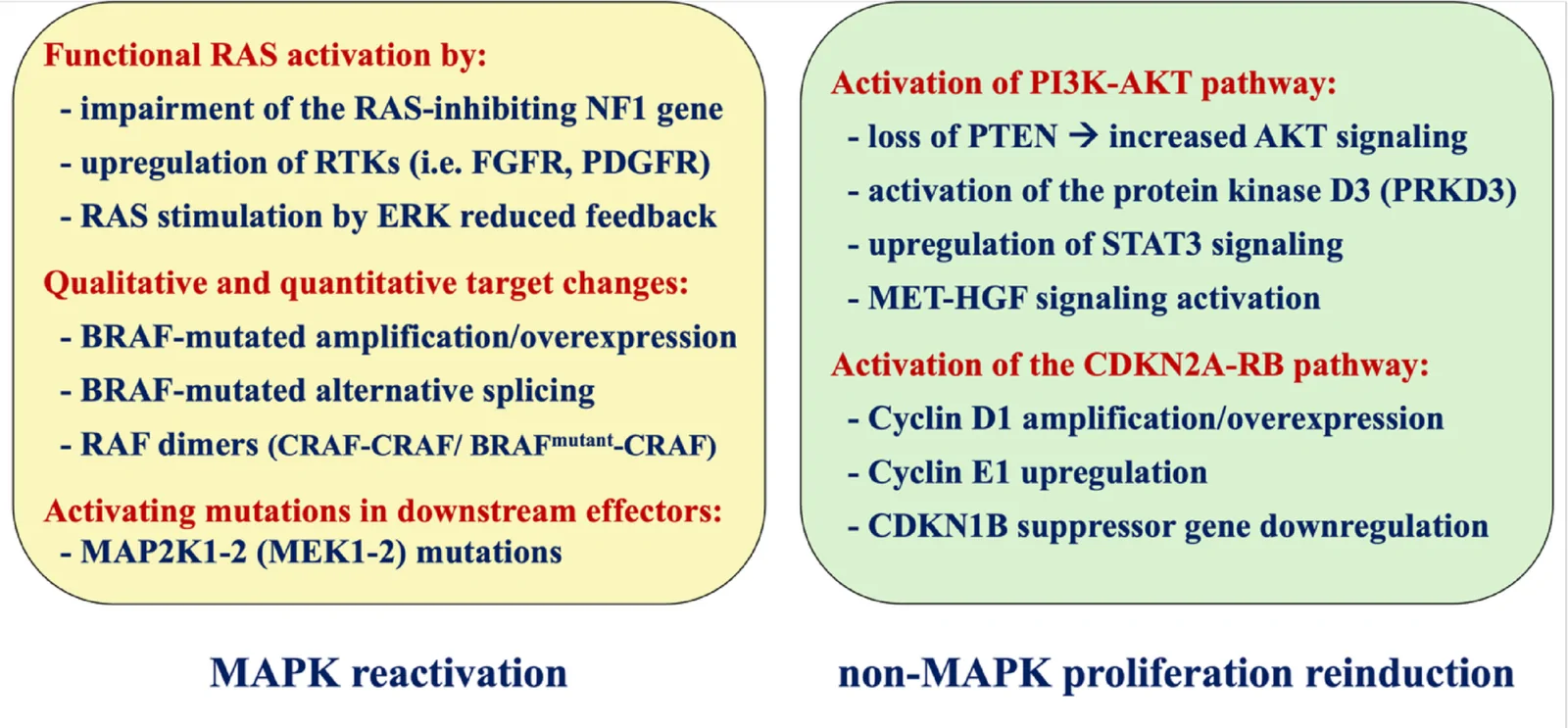
Main mechanisms of resistance to BRAF and MEK inhibitors. RTKs, receptor tyrosine kinases

Acquired resistance frequently involves reactivation of the MAPK pathway through BRAF-dependent mechanisms (secondary BRAF mutations, splice variants, epigenetic changes, or gene amplification) or BRAF-independent alterations occurring upstream (RAS activation or KRAS mutations) or downstream (MAP2K1-2/ MEK1-2 mutations) of BRAF [32]. Additionally, bypass mechanisms have been described, most notably involving the PI3K/AKT, CDKN2A-RB, and FGFR signaling pathways [109, 110]. The Fig. 4 summarizes the different molecular changes involved in such a resistance, grouping events in two separate scenarios: reactivation of the MAPK pathway and reinduction of cell proliferation through activation of pathways outside the MAPK cascade.

The occurrence of concurrent gene mutations in BRAF-mutated NSCLC patients treated with BRAF/MEK inhibitors appears not to be associated with a worse outcome [84]. Overall, the emergence of resistance represents a major challenge in the treatment of patients with BRAF-mutant NSCLC, as it limits the durability of clinical responses and reduces the long-term efficacy of targeted therapies, highlighting the need to develop next-generation agents capable of overcoming both resistance mechanisms and treatment-related toxicities, while potentially providing enhanced antitumor efficacy and more durable clinical benefit.

### Issues for Research: New Players and Future Perspectives

Several ongoing trials are currently investigating novel targeted therapies, including agents with broader biological activity such as pan-RAF inhibitors, pan-RAF/MEK inhibitors, and MAPK pathway inhibitors, which may provide more durable responses and improved ability to overcome resistance mechanisms (Table 2).

**Table 2:** Ongoing clinical trials investigating novel agents and treatment schemes in BRAF – mutated NSCLC

Agents/treatments	Molecular targets	Phase	Status	Trial code
*Targeted therapies*				
Cobimetinib, Vamurafenib	BRAF	2/3	Active, not recruiting	NCT03178552
FORE8394	BRAF	1	Terminated	NCT06270082
XP-102 ± Trametinib	panRAF ± MEK	1/2	Not yet recruiting	NCT05275374
KIN-2787 ± binimetinib	panRAF ± MEK	1	Active, not recruiting	NCT04913285
NST-628	panRAF/MEK	1	Recruiting	NCT06326411
IMM-1-104 ± dabrafenib	BRAF ± MEK	1/2	Active, not recruiting	NCT05585320
IK-595	MAPK	1	Terminated	NCT06270082
PF-07799544	MEK	1	Recruiting	NCT05538130
PF-07799933 ± binimetinib	panRAF ± MEK	1	Recruiting	NCT05355701
S241656	Pan-RAF	2/3	Recruiting	NCT05786924
DCC-3084	BRAF/CRAF	1/2	Terminated	NCT06287463
Dabrafenib ± Trametinib	BRAF ± MEK	4	Recruiting	NCT03340506
Dabrafenib +Trametinib neoadiuvant	BRAF + MEK	2	Recruiting	NCT06563999
Dabrafenib + Trametinib + Aspirin	BRAF + MEK	Observational	Not yet recruiting	NCT05988697
*Immunotherapy (± targeted)*				
Atezolizumab + Cobimetinib	BRAF + PD-L1	2	Active, not recruiting	NCT03600701
IMM-1-104 ± pembrolizumab	MEK ± PD-1	1/2	Active, not recruiting	NCT05585320
Recombinant EphB4-HSA Fusion Protein + pembrolizumab	BRAF + PD-1	1/2	Completed	NCT03049618
Toripalimab + Chemotherapy neoadiuvant	PD-1	2	Recruiting	NCT05800340
Immunotherapy	PD-1, PD-L1, CTLA4	Retrospective	Recruiting	NCT04777175
*Other selected*				
Sacituzumab tirumotecan vs. chemotherapy	TROP2	3	Recruiting	NCT06074588

Cobimetinib, a MEK inhibitor previously used in combination with vemurafenib for BRAF-mutated melanoma and other solid tumors, has recently been evaluated in combination with ABM-1310, an oral BRAF V600 inhibitor with high brain barrier penetration [113]. In this first-in-human phase I study, ABM-1310 was evaluated in patients with BRAF V600–mutated advanced solid tumors, including brain metastases and primary CNS tumors. The drug, as monotherapy or combined with cobimetinib, showed a manageable safety profile, dose-proportional pharmacokinetics, and promising activity, with ORRs of 12% overall and 23% in CNS tumors, supporting further investigation [113]. Cobimetinib is currently under investigation in B-FAST study (NCT03178552) and in a phase 2 study in association with atezolizumab, an anti-PD-L1 immunotherapy agent (NCT03600701) (Table 2).

Regarding immunotherapy, we previously highlighted the relative lack of prospective clinical trials, particularly in the first-line setting. Aside from the previously mentioned study evaluating the combination of atezolizumab plus cobimetinib, two additional trials are of particular interest: one investigating IMM-1-104 (a MEK1/2 inhibitor) with or without pembrolizumab (NCT05585320), and another evaluating the combination of recombinant EphB4-HSA fusion protein plus pembrolizumab (NCT03049618, Table 2). A relative paucity of studies also exists regarding the use of targeted therapies or immunotherapy in the neoadjuvant setting. A phase II trial is currently evaluating neoadjuvant toripalimab in combination with chemotherapy (NCT05800340); however, additional studies will be needed in the future to further explore this therapeutic approach.

A phase III clinical trial is currently evaluating an anti‑TROP‑2 antibody–drug conjugate (ADC) versus chemotherapy in NSCLC patients harboring EGFR mutations or other genetic alterations, including BRAF mutations (NCT06074588). ADCs are targeted therapeutics that link a tumor-specific monoclonal antibody to a potent cytotoxic payload via a chemical linker, allowing selective delivery to malignant cells while sparing normal tissues; they have demonstrated clinical efficacy across both hematologic and solid tumors and represent a rapidly expanding area of oncology drug development [114]. Similarly, chimeric antigen receptor (CAR) T‑cell therapies have shown encouraging results in preclinical studies of NSCLC and represent a promising avenue for future research, including in patients with driver genetic alterations. CAR‑T cells are genetically engineered to express chimeric antigen receptors that specifically recognize tumor-associated antigens, enabling targeted cytotoxic activity against malignant cells [115]. In addition, CAR‑NK and CAR‑macrophage (CAR‑M) therapies are emerging as complementary cellular immunotherapy approaches, leveraging natural killer cells or macrophages engineered to recognize tumor antigens, and may offer distinct advantages in safety, tumor infiltration, and overcoming immunosuppressive tumor microenvironments.

## Conclusions

In summary, activating BRAF mutations are found in approximately 3–4% of NSCLC patients, the majority being the V600E variant, which permits treatment with approved BRAF inhibitors, either as monotherapy or in combination with MEK inhibitors. Evidence supporting the use of immunotherapy remains limited, although it may be appropriate for selected subsets of BRAF-mutated patients. Despite notable advances in targeted therapies and immunotherapy for BRAF‑mutant NSCLC, treatment resistance and limited clinical data, particularly in first line and neoadjuvant settings, remain major challenges. Several ongoing studies are currently evaluating novel agents with broader biological activity and various drug combinations. Emerging strategies, including next-generation MAPK inhibitors, ADCs, and engineered cellular therapies such as CAR‑T, CAR‑NK, and CAR‑M, offer promising avenues to enhance efficacy, overcome resistance, and expand therapeutic options for these patients.

## Data Availability

No datasets were generated or analysed during the current study.

## Abbreviations

NSCLC: Non-small cell lung cancer
SCLC: Small cell lung cancer
ADC: Adenocarcinoma
SCC: Squamous cell cancer
PD-L1: Programmed death-ligand 1
ICIs: Immune checkpoint inhibitors
IRR: Independent radiology review
COSMIC: Catalogue of Somatic Mutations in Cancer
TCGA: The Cancer Genome Atlas
PTC: Papillary thyroid carcinoma
RTKs: Receptor tyrosine kinases
MAPK: Mitogen-activated protein kinase
MEK: Mitogen-activated extracellular signal regulated kinase
ERK: Extracellular signal-related kinase
MSI: Microsatellite instability
NCCN: National Comprehensive Cancer Network
NGS: Next-generation sequencing
PCR: Polymerase chain reaction
ctDNA: Circulating tumor DNA
ISLB: International Society for Liquid Biopsy
MRD: Minimal residual disease
PFS: Progression-free survival
SAEs: Serious adverse events
ORR: Objective response rate
OS: Overall survival
ADC: Antibody-Drug Conjugate
